# Dog bites as a zoonotic risk in Ecuador: Need for the implementation of a One Health approach

**DOI:** 10.1016/j.onehlt.2023.100544

**Published:** 2023-04-19

**Authors:** G. Joselyn Calderón, Silvia Poveda, Ariana León Sosa, Naomi Mora, Manel López Bejar, Solón Alberto Orlando, Miguel Angel Garcia-Bereguiain

**Affiliations:** a Instituto Nacional de Investigación en Salud Pública “Leopoldo Izquieta Pérez”, Dirección Técnica de Investigación, Desarrollo e Innovación, Guayaquil, Ecuador; b Universidad Agraria del Ecuador, Guayaquil, Ecuador; c Universidad Autónoma de Barcelona, Spain; d One Health Research Group, Universidad de Las Americas, Quito, Ecuador; e Universidad Espiritu Santo, Guayaquil, Ecuador

**Keywords:** Dog bites, Rabies, Canines, Dogs, One Health, Ecuador

## Abstract

Rabies is a viral zoonotic disease that can infect all mammals and the main route of transmission to human is attributed to dog bites. Due to the limited information available about the rabies vaccination coverage, although Ecuador is supposed to be free of rabies, we conducted a retrospective study of the epidemiological surveillance records on the notification of dog attacks to humans in Guayaquil, the most populated city in Ecuador. The results showed an annual incidence rate of 105.6 dog bites per 100,000 inhabitants, where the most affected anatomical parts are the lower extremities; individuals from 1 to 14 years of age were the most affected age group (IC95% 1.42–1.92; *p* < 0.001). As for the severity of the wounds, most of them (65%) were mild. Moreover, 25% of the dogs were free roaming ones, and only 43% of the dogs with owner had a complete vaccination scheme against rabies virus. We found a important dog attack rate in Guayaquil city and more than half of the dogs involved were not vaccinated against rabies. Under a potential scenario of rabies circulation in canine population, there would be a serious risk for rabies transmission to humans. Hence, it is important to reinforce rabies surveillance and vaccination programs aligned to the One Health concept to manage this public health issue.

## 1 Introduction

The COVID-19 pandemic has shown the need for a multidisciplinary approach in the care of emerging and re-emerging diseases, thus strengthening the “One Health” strategy. It is estimated that 60% of emerging diseases in the world are zoonotic diseases (1). Rabies is a viral zoonotic disease transmitted by the bite of infected canines, and cause around 60,000 deaths worldwide annually (1). Although human rabies is a preventable disease, it is not the only source of public health concern, as dog bite wounds are prone to local and systemic bacterial infections (2). Incorrect cleaning and delays in the treatment of these lacerations can promote the appearance of severe infections causing irreversible physical damage (3,4).

Transmission of zoonotic diseases through dog bites is a public health issue in low and middle income countries where the presence of free roaming dogs is usual (5), not only in rural areas (6) but also in the main cities (7). For instance, the free roaming dog population in Guayaquil (the most populated city of Ecuador) has been estimated to be bigger than 30,000 (7). Moreover, in the context of tropical countries of the Americas like Ecuador, urban fauna also includes wild animals like raccoons and opossums as cities are surrounded by the tropical forest and there is a great diversity of bats including vampire bats. Also, illegal trafficking of wild species to be kept as pets is not rare (6,8). So, the exposure of dogs and cats, or even humans, to potential reservoirs of rabies is a permanent threat for rabies outbreaks.

In 1996, Ecuador experienced the highest rate of rabies per capita in the Americas, with 65 human cases and 1175 canine cases (9). Since then, an effective vaccination program reduced the rabies cases, and according to the Ministry of Health of Ecuador, the last cases of human rabies transmitted from dogs were in 2006, and the last cases reported of canine rabies were in 2011 (9,10). However, there was a rabies outbreak causing 11 deaths associated to bat bites in the Amazonian Region of Ecuador in 2011 (9). Despite this potential threat for rabies transmission from wild reservoirs either to human or dogs, the Ministry of Health of Ecuador is currently working in the certification of Ecuador as a human rabies free country (10). Nevertheless, the Centers for Disease Control and Prevention from USA includes Ecuador within the countries with risk of rabies transmission, and recommend vaccination prior to travel (11). Moreover, there is not public available updated information about rabies vaccination coverage in dogs in Ecuador. Nevertheless, the reemergence of rabies in rabies-free areas is always a threat as long as free roaming dogs and cats are present, and urban outbreaks of rabies have been recently described in South America ([12], [13], [14]).

The aim of this study was to evaluate the potential risk of rabies transmission to human associated to dog bites in the city of Guayaquil, Ecuador. The surveillance information of dog bites to humans in this city for 2013–2016 was included in this study, although this problem currently persists as the population of free roaming dogs is far for declining (7) and the dogs attacks has created a recent social alarm as it was broadcasted by local media (15). The goal of this report is to rise concern about this public health issue that should be address from the perspective of the One Health concept.

## 2 Methods

### 2.1 Study design

A retrospective descriptive study was carried out, collecting information on dog bites to people from epidemiological files established by the Ministry of Public Health in Ecuador. These data came from 14 first-level health care centers, which were classified according to their location in 2 zones: north and south of the city of Guayaquil and during two study periods including years 2013 to 2016 (Table 1).

**Table 1:** Primary care centers included in the study and collection time.

Study period	First level care centers in the South Zone of Guayaquil	First level care centers in the North Zone of Guayaquil
**Period 1:** From January 2013 to April 2014	➔ Santiago de Guayaquil ➔ 28 de Febrero ➔ Mariscal Sucre ➔ La Laguna ➔ San Francisco de Asís ➔ San José ➔ Héroes del 41	➔ Centro de salud materno Bastión popular ➔ Pascuales ➔ Las Orquídeas ➔ Los Vergeles ➔ Bastión Popular # 1 ➔ Bastión Popular # 2 ➔ Bastión Popular # 3
**Period 2**: From May 2014 to July 2016 in the north zone	⁎N/A	

⁎ N/A: not applicable.

Regarding the data collected, the following patient factors were analyzed: age, sex, severity of the injury, location of the wound; while the aggressor canine factors included: sex of the animal, rabies vaccination status, canine with owner - without owner. Subsequently, we performed a descriptive analysis of the data by calculating both frequency and association measures using the Epi Info TM 7 - R version 2.47. Fig. 2, Fig. 5 were created with BioRender.

### 2.2 Ethics statement

The evaluation of a Human Research Ethics Committee was not required given the retrospective nature of the study, which consisted of non-experimental research with secondary data previously anonymized. Nevertheless, the Board of the National Institute of Public Health (“Instituto Nacional de Salud Pública e Investigación “Leopoldo Izquieta Pérez”) granted the authorization to carry out this study in accordance with the policies established by the Ecuadorian government.

## 3 Results

### 3.1 Incident rate of dog bites during the study period 2013–2016

**Fig. 1:**
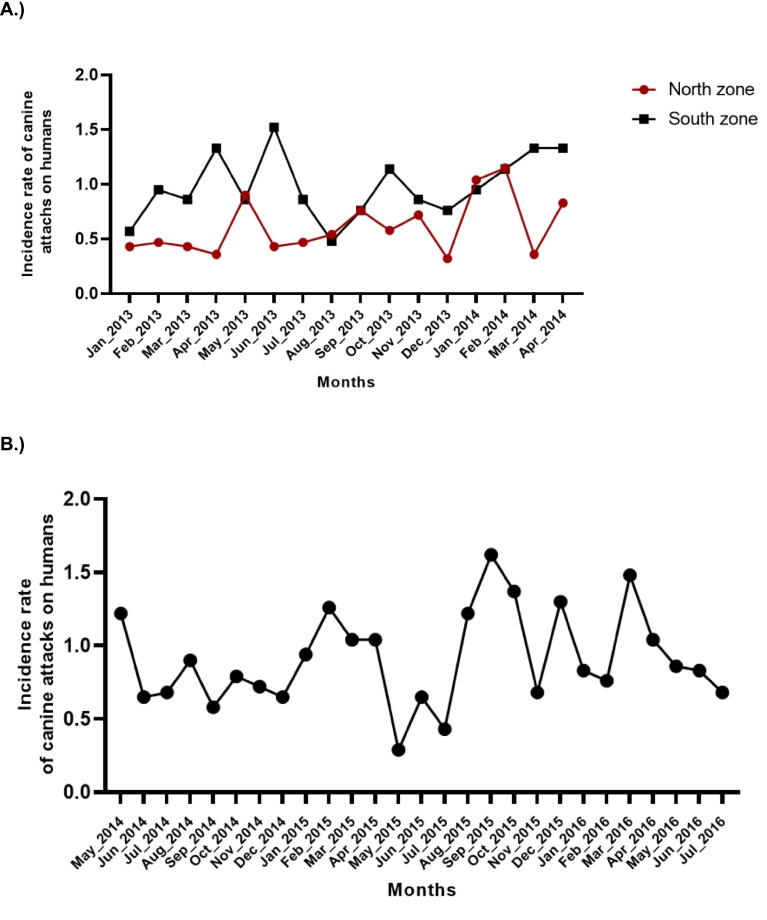
Comparative analysis of canine attacks in the areas studied during (a) the first (North and South Zone) and (b) second study period (North zone).

During the study period of 2013 to 2016, a total of 1118 dog bites to humans were reported. The monthly average rate obtained was of 0.79 and 0.90 cases per 100,000 inhabitants for the periods January 2013–April 2014 and May 2014–July 2016 (Fig. 1).

### 3.2 Type and severity of dog bites

**Fig. 2:**
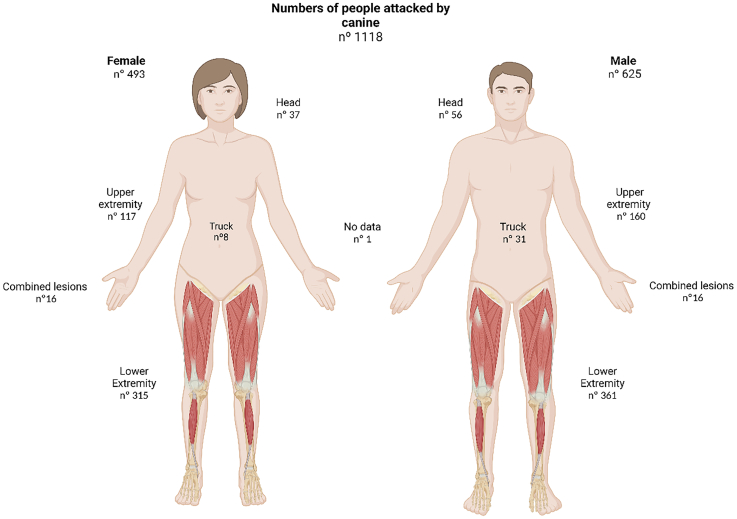
Distribution of bites in areas of the body of humans attacked by dogs included in this study.

**Fig. 3:**
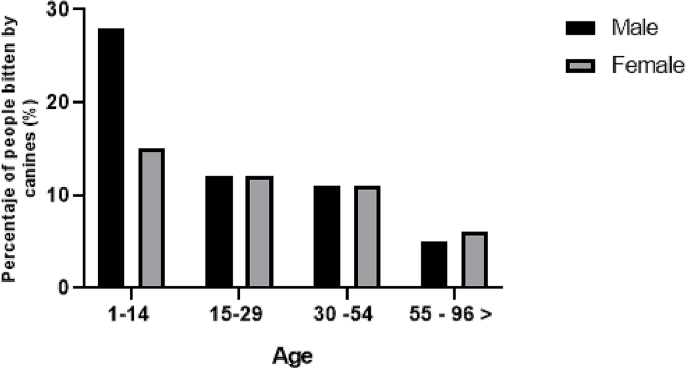
Age and sex distribution of humans suffering dog bites included in this study.

When categorizing dog bites by body parts, 60.45% of the bites affected lower extremities and 39.54% affected the rest of the body (Fig. 2). Regarding the age and sex distribution of human suffering dog bites (Fig. 3), males aged 1 to 14 years were more susceptible to canine attacks, representing 28% of the overall attacks (*p* < 0.001).

**Fig. 4:**
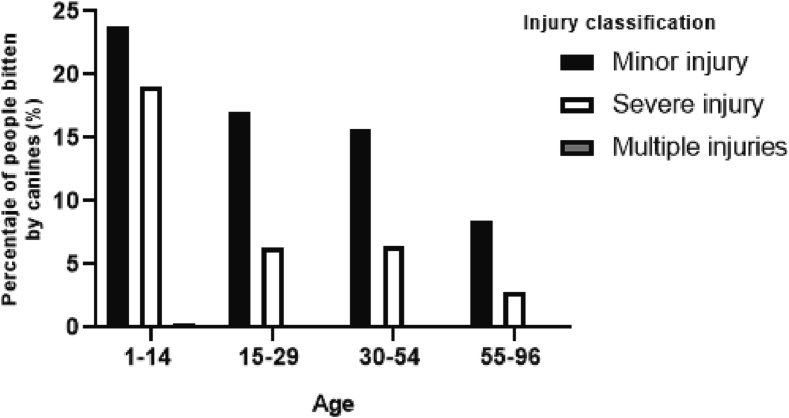
Percentage of people bitten by dogs per severity of injury and age group.

Regarding the severity of bites, mild abrasions were the most common in all age groups, representing 65% (727). Severe lesions were more common in age group 1 to 14 years, accounting for 19% (212) of the total bites (p < 0.001) (Fig. 4).

### 3.3 Characterization of dogs involved in the attacks to humans

**Fig. 5:**
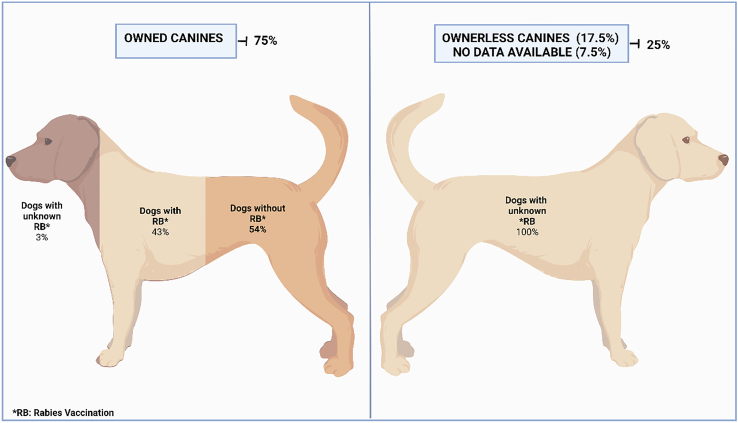
Ownership and vaccination status of the canines involved in attacks to humans included in this study.

Male dogs attacked 3.6 times more than females. Also, 75% (838) of the attacking dogs had owners. Among those, 54% (452) were not vaccinated against rabies The remaining 25% (280) of dogs had no owners and most likely were not vaccinated (Fig. 5).

## 4 Discussion

In this study, we evaluated dog attacks to human in the city of Guayaquil as a potential risk for rabies outbreaks. During the study period, there were 1118 cases of dog bites in the two areas included in the study in the city of Guayaquil. However, this number may be higher, as less serious injuries could not be reported, particularly in the context of low and middle income countries like Ecuador where free healthcare assistance is not always guaranteed. This is particularly important for neglected communities living under poverty in some areas of the city of Guayaquil where the presence of free roaming dogs is higher (7). Hence, it is important that public health authorities define more accurate strategies regarding dog attacks, including educating the population about the serious health risk associated to dog bites.

Regarding the the sex of bitten people, it was observed that males were attacked more frequently than females, in agreement with other studies ([15], [16], [17], [18], [19], [20]). With respect to age, the most affected group was from 1 to 14 years old. These results were similar to other studies where the most affected age group were kids (17,21), although other reports found more attacks to adults and elderly (19,22). Regarding the anatomical location of the wounds, the most affected parts were lower extremities, more accessible to the dog. Upper extremities were the second region with the highest number of bite injuries, generally used for protection during the attack ([23], [24], [25], [26]). Mild abrasions represented 65% of the injuries which were reported in all age groups. However, serious injuries generally occur in ages 1–14 years, thus, suggesting that children are at greater risk of suffering serious.

Male dogs attacked more frequently than females, in agreement with data published in other studies. This could be associated to the effect of androgens on the dog behavior when not neutered as usually happened with free roaming dogs that where highly represented in our study population (27,28). These results endorsed the importance of sterilization campaigns for free roaming dogs but also for owned dogs not only as a way to control population growth but also to prevent attacks to humans.

It is important to notice that 25% of the dogs responsible for attacks were free roaming dogs in our study. It is plausible to assume that those dogs were not vaccinated against rabies. Moreover, among the 75% of dogs with owners included in the study, 54% were not vaccinated against rabies. Thus, in the most populated city of Ecuador, Guayaquil, more than half of the dogs involved in humans attacks were not immunized against rabies. This is a worrying scenario, considering that rabies outbreaks affecting humans have happened in Ecuador recently, associated to bat bites and resulting in several deaths (8). Additionally, urban fauna also includes wild mammals like raccoons and opossums as Guayaquil city is close to mangroves and tropical forest, and illegal trafficking of wild species to be kept as pets is not rare (6,8), increasing the risk of exposure of dogs and cats, or even human, to wild reservoir of rabies. The existence of wild reservoirs for rabies in a context of low vaccination rate in dogs represents a threat for canine rabies outbreaks in Ecuador. Moreover, considering the high population of free roaming dogs in Ecuador (7), and the occurrence of dog bites as reported in this study, the risk of human rabies outbreaks like happened in Ecuador in the 90s (9) is still a public health concern.

While the Ministry of Health is working in the rabies-free status for Ecuador (9), the Center for Diseases Control and Prevention from USA still considered Ecuador as risk country for rabies (10). A canine rabies surveillance and vaccination program including periodic reports and encouraging public health authorities to publish their results would help to promote a better perception of the rabies epidemiology in Ecuador by international public health agencies. Although a national massive rabies vaccination started in 2016 with the goal of immunized 90% of cats and dogs, there are not published reports about the canine population covered and if free roaming dogs are included in those campaigns. Moreover, reemergence of rabies in rabies-free urban areas of Peru and Argentina have been recently described ([12], [13], [14]). In Peru, starting from Arequipa city in 2015, outbreaks of rabies in free roaming urban dogs have expanded to several cities till 2019 (12,13). In Argentina, a fatal case of human rabies transmitted by a feral cat attack previously infected by bats was described in 2021 (14). Under this scenario, the threat of rabies reemergence in Ecuador should be considered by public health authorities and public health policies to prevent it should be reinforced.

Considering that dog ownership is legally the responsibility of local governments (Municipalities) in Ecuador, but canine rabies vaccination program and the care of affected patients is the responsibility of the central government, it is worth recommending a holistic multidisciplinary approach anchored in the One Health concept among all the stake holders involved in this public health issue.

In conclusion, despite human or canine rabies cases have not been reported for several years in Ecuador, the presence of wild reservoir for rabies, the lack of information regarding the canine rabies vaccination program and the high population of free roaming dogs, makes the frequent dog bites to humans reported in this study a threat for human rabies outbreaks. A One Health approach to solve this public health issue is necessary promoting access to sterilization and rabies vaccination to owned dog and cats, rabies surveillance of free roaming dogs and cats and other wild mammals urban populations; and education policies to rise concern about the public health risk of wildlife trafficking.

## Author contributions

All authors contributed to sample collection and data analysis. JCG, MAGB and SAO wrote the manuscript. All the authors critically reviewed and approved the manuscript.

## Declaration of Competing Interest

The authors declare that they have no known competing financial interests or personal relationships that could have appeared to influence the work reported in this paper.

## Data Availability

Data will be made available on request.
